# Accelerated and natural carbonation of concrete with high volumes of fly ash: chemical, mineralogical and microstructural effects

**DOI:** 10.1098/rsos.181665

**Published:** 2019-01-16

**Authors:** Philip Van den Heede, Mieke De Schepper, Nele De Belie

**Affiliations:** Magnel Laboratory for Concrete research, Department of Structural Engineering, Ghent University, Tech Lane Ghent Science Park, Campus A, Technologiepark Zwijnaarde 60, 9052 Ghent, Belgium

**Keywords:** carbonation, high-volume fly ash, silica fume, thermogravimetric analysis, X-ray diffraction, mercury intrusion porosimetry

## Abstract

Today, a rather poor carbonation resistance is being reported for high-volume fly ash (HVFA) binder systems. This conclusion is usually drawn from accelerated carbonation experiments conducted at CO_2_ levels that highly exceed the natural atmospheric CO_2_ concentration of 0.03–0.04%. However, such accelerated test conditions may change the chemistry of the carbonation reaction (and the resulting amount of CH and C–S–H carbonation), the nature of the mineralogical phases formed (stable calcite versus metastable vaterite, aragonite) and the resulting porosity and pore size distribution of the microstructure after carbonation. In this paper, these phenomena were studied on HVFA and fly ash + silica fume (FA + SF) pastes after exposure to 0.03–0.04%, 1% and 10% CO_2_ using thermogravimetric analysis, quantitative X-ray diffraction and mercury intrusion porosimetry. It was found that none of these techniques unambiguously revealed the reason for significantly underestimating carbonation rates at 1% CO_2_ from colorimetric carbonation test results obtained after exposure to 10% CO_2_ that were implemented in a conversion formula that solely accounts for the differences in CO_2_ concentration. Possibly, excess water production due to carbonation at too high CO_2_ levels with a pore blocking effect and a diminished solubility for CO_2_ plays an important role in this.

## 1. Introduction

Fly ash (FA) already has a long history of being used as partial replacement of ordinary Portland cement (OPC) in concrete. When focusing on high-volume fly ash (HVFA) concrete, special attention should go to environments subject to atmospheric CO_2_ ingress and carbonation-induced steel corrosion. In OPC concrete, CO_2_ dissolves in the pore solution to form carbonic acid which reacts with Ca(OH)_2_ (CH) and calcium silicate hydrates (C–S–H) in the cement paste, forming mainly CaCO_3_. Although CH carbonation results in a more dense microstructure due to the fact that the volume of the calcite formed is 11–12% greater than the volume of the original CH [1], the related drop in pore fluid alkalinity can disrupt the protective passivation layer on embedded steel and cause active corrosion [2]. In HVFA concrete, the pozzolanic hydration reaction of FA also consumes CH [3]. As a result, less CH is available and the carbonation front moves inwards faster. Moreover, the lower CH availability will result in more C–S–H carbonation [1]. In contrast with CH carbonation, C–S–H carbonation coarsens the pore structure. The removal of interlayer calcium creates an excess of negative charges, which are balanced through subsequent formation of Si–OH groups. Condensation of the neighbouring Si–OH groups to Si–OH–Si linkages then forms silica gel [4]. It increases the mean silicate chain length and forms bridges between neighbouring regions. As a result, these regions are being pulled together leading to shrinkage. In other words, the polymerization of the silicate chains in C–S–H may cause a volumetric decrease as well as cracking and a coarsening of the pore structure. Also note that CH carbonation precipitates mainly well-crystallized calcite, while its amorphous and metastable, crystalline polymorphs (vaterite and aragonite) are more likely the result of C–S–H carbonation [5].

According to Castellote *et al*. [6], at least part of the shrinkage due to the carbonation of C–S–H observed by Borges *et al*. [1] may also be attributed to the fact that the carbonation test was performed in an atmosphere with 5% CO_2_ instead of the proposed maximum value of 3% that normally does not alter the natural carbonation process. Performing carbonation tests at a CO_2_ concentration above 3% may overestimate the carbonation of C–S–H, the coarsening of the microstructure and thus the measured carbonation depths and rates that result from it. It is important to realize this because the higher susceptibility of FA binders to carbonation is usually concluded from accelerated tests at high CO_2_ levels. The applied CO_2_ concentration in carbonation-related scientific literature can range from 1% to 100% [7]. For OPC binder systems, it has already been demonstrated that accelerated test conditions could alter the carbonation process, yet for HVFA binder systems this was studied less. In this paper, the chemical, mineralogical and microstructural effects of accelerated carbonation at 10% and 1% CO_2_ as opposed to natural carbonation at 0.03–0.04% CO_2_ have been investigated for pastes with a 50% and 40%+10% replacement level of the OPC by FA and fly ash + silica fume (FA + SF), respectively. CH to C–S–H carbonation ratios were quantified from thermogravimetric analyses (TGA). Related mineralogical phases were identified and quantified with X-ray diffraction (XRD) and subsequent Rietveld analysis. Changes in porosity and pore size distribution were determined via mercury intrusion porosimetry (MIP).

## 2. Material and methods

### 2.1. Concrete and paste mixtures

In total, three concrete mixtures were manufactured (table 1). Mixture T(0.55) is an OPC concrete composition with a minimum cement content and a maximum water-to-cement (W/C) ratio conforming to NBN B15-001 [8] for exposure class XC3, a moderately humid environment with exposure to carbonation-induced steel corrosion. A common example of such an environment is exterior concrete sheltered from rain. For the other two compositions, 50% of the total binder (B) content consisted of supplementary cementitious material (SCM). Mixture F50 counts as a HVFA concrete because half of the total binder content consisted of pozzolanic FA. To compensate for the rather slow hydration reaction of the FA, a higher total binder content of 450 kg m^−2^ and lower water-to-binder (W/B) ratio (0.35) were applied. As such, quite a high early age strength performance could still be achieved. The strength class of the material (= C40/50), which is based on the 28-day characteristic compressive strength, proves this. To improve the early age strength performance without increasing the total binder content too much, SF could be introduced as third powder in the binder system. This was done for composition F40SF10 in which the total binder content consisted of 50% of Portland cement, 40% of FA and 10% of SF. With a total binder content of only 340 kg m^−3^, a strength class of no less than C50/60 could be achieved. It should be noted that the use of a lower W/B ratio for mixtures F50 and F40SF10 (0.35 versus 0.55) in view of achieving an adequate early age strength performance normally also has consequences for their carbonation behaviour. A lower W/B ratio normally contributes to a lower porosity and permeability to CO_2_, hence a lower carbonation depth and rate, and thus an increased carbonation resistance. Nonetheless, lowering the W/B ratio for F50 and F40SF10 does not guarantee a carbonation resistance similar to the one of the OPC reference T(0.55) with a W/C ratio of 0.55. Apart from the porosity-related permeability to CO_2_, the low CO_2_ buffering capacity in the presence of pozzolanic FA also plays a major role in the binder's carbonation resistance. To what extent the lowering of the W/B ratio will have compensated for a faster CO_2_ ingress because of the lower CO_2_ buffering capacity, will follow from the data presented in §3.1.

**Table 1.** Concrete mixture proportions.

	T(0.55)	F50	F40SF10
sand (kg m^−3^)	715	645	791
gravel (kg m^−3^)	1188	1071	1141
CEM I 52.5 N (kg m^−3^)	300	225	170
fly ash (FA) (kg m^−3^)	0	225	136
silica fume (SF) (kg m^−3^)	0	0	34
water (kg m^−3^)	165	157.5	119
superplasticizer (ml kg^−1^ B)	2	5	12
W/B	0.55	0.35	0.35
FA/B	0	50	40
SF/B	0	0	10
slump^a^	S4	S5	S4
strength class^b^	C30/37	C40/50	C50/60

^a^S1 (10–40 mm), S2 (50–90 mm), S3 (100–150 mm), S4 (160–210 mm), S5 (≥ 220 mm).

^b^Based on the 5% characteristic (compressive strength) value.

Changes in chemistry, mineralogy, microstructure induced by carbonation at 1% and 10% were studied on pastes with the same binder composition and W/B ratios as concrete mixtures T(0.55), F50 and F40SF10. In terms of carbonation behaviour, these pastes should be representative for the paste volume present in the corresponding concrete mixtures. In Hermida *et al*. [9], it was experimentally proved that for a paste volume varying between ± 200–360 l m^−3^ of concrete, the carbonation depth always remains in the same range. This is an indication that the effect of the interfacial transition zone on the carbonation resistance is negligible. As such, it should be justified to say that concrete and paste with a composition similar to the paste volume of that concrete should show a similar carbonation behaviour.

### 2.2. Sample preparation and preconditioning

Per concrete mixture, 24 cubes with a 100 mm side were cast and optimally cured at 20°C and 95% relative humidity (RH) for 28 days. An impermeable coating was applied on 5 of the 6 cube surfaces to ensure a unidirectional flow of CO_2_ throughout the samples during the carbonation experiments. The untreated side was always a cast surface of the cube.

The pastes were cast in sealable cylindrical moulds (Ø: 46 mm, height: 50 mm) that were kept on rotating stands until the next day in order to prevent bleeding and segregation. Then, the cylinders were cured under water at 20 ± 2°C until they reached the age of 28 days. Once surface dry, the upper and lower surfaces of the paste cylinders were sealed with an impermeable aluminium tape leaving only the mantle surface for exposure.

### 2.3. Carbonation tests

Per mixture, half of the cubes was subjected to an accelerated carbonation test at 10% CO_2_, 20°C and 60% RH, while the other half was kept in a carbonation cabinet at 1% CO_2_, 20°C and 60% RH for a similar test. According to da Silva *et al*. [10], a concentration of 1% CO_2_ develops the same reaction products as a normal atmosphere at 0.03–0.04% CO_2_ and can thus be considered as a more or less natural carbonation process. After 4, 8, 12 and 16 weeks of exposure, three cubes per mixture were split to enable colorimetric carbonation assessment on fractured surfaces using the phenolphthalein colour indicator. Per fractured surface treated with phenolphthalein, nine different measurements (one every 10 mm) were done, and this to the nearest millimetre. After spraying the 1% phenolphthalein solution onto the concrete slices or fractured surfaces, the carbonated area will be colourless, while the non-carbonated area will be purple.

With respect to the paste mixtures, two cylinders per mixture were stored in the carbonation cabinets at 1% and 10% CO_2_ for 12 weeks. Some additional cylinders were crushed into very small pieces (Ø: 1–3 mm). They were kept in a normal environment at 20°C and 60% RH until they were carbonated in a fully natural way. In such an atmosphere, the CO_2_ concentration is normally around 0.03–0.04%. Another set of cylinders was continuously cured in water for 182 days.

### 2.4. Carbonation rate and resistance

For each concrete mixture tested, the measured carbonation depths (in millimetre) with the phenolphthalein colour indicator were plotted as function of the square root of the exposure time *t* (in weeks) to determine an experimental (accelerated) carbonation coefficient *A*_acce_ (in mm/√weeks). The carbonation rate obtained cannot be considered as a realistic one, because a CO_2_ concentration of 10% exceeds the natural CO_2_ concentration in air (0.03–0.04%) by far. To obtain a first estimation of the corresponding carbonation rate under field conditions from accelerated carbonation tests performed at 10% CO_2_, Audenaert [11] used a conversion formula that expresses the ratio of the accelerated and field carbonation coefficients (*A*_acce_ and *A*_field_) in terms of their corresponding CO_2_ concentrations *c*_acce_ and *c*_field_. The same formula (equation (2.1)) was used in this research.

2.1 $$\frac{A_{\mathrm{acce}}}{A_{\mathrm{field}}}=\frac{\sqrt{c_{\mathrm{acce}}}}{\sqrt{c_{\mathrm{field}}}}.$$

Note that Sisomphon & Franke [12] used a very similar conversion formula. Only the CO_2_ concentration of their accelerated carbonation test was different (3% instead of 10%). As the literature indicates that 3% CO_2_ could be the maximum allowable CO_2_ concentration for an accelerated carbonation test (see §1), one could conclude that equation (2.1) may not be applicable for all values of *c*_acce_. We therefore calculated *A*_acce_ of one HVFA mixture (F50), one FA + SF mixture (F40SF10) and one OPC reference (T(0.55)) from both the outcome of the carbonation test performed at 10% CO_2_ and 1% CO_2_ and also converted the *A* values for 10% CO_2_ to *A* values representative for 1% CO_2_ using equation (2.1) in which the 1% values were assumed equal to the field values.

According to Visser [13], there is another way to see whether accelerating the carbonation test by increasing the CO_2_ concentration may lead to unwanted effects, such as an important over- or underestimation of the concrete's service life when based upon these kinds of tests. The evaluation procedure follows directly from the well-known square-root-time relation for carbonation (equation (2.2)).

2.2 $$x_{c}=\sqrt{\frac{2\cdot D_{c}\cdot c_{s}\cdot t}{a_{c}}}=\sqrt{\frac{2\cdot c_{s}\cdot t}{R_{\mathrm{carb}}}},$$

with *x*_c_, the carbonation depth (m); *D*_c_, the diffusion coefficient of CO_2_ (m^2^ s^−1^); *c*_s_, the CO_2_ concentration at the concrete surface (kg m^−3^); *a*_c_, the amount of carbonatable material per unit volume (kg m^−3^); *t*, the time (*s*) and *R*_carb_ (= *a*_c_/*D*_c_), the carbonation resistance ((kg m^−3^)/(m^2^ s^−1^)). When plotting the measured carbonation depths during the accelerated carbonation tests at 1% and 10% CO_2_ as a function of √(2 · *c*_s_ · *t*), the slope of the linear trends obtained should be equal to √(1/*R*_carb_). As *R*_carb_ is a material variable which is normally independent of the applied CO_2_ concentration, the slopes should be similar for the two carbonation tests. If not, some other unwanted effects must have occurred.

### 2.5. Thermogravimetric analysis

Powdery samples were collected from the paste cylinders before and after exposure to increased CO_2_ concentrations (1% and 10%) in the carbonation cabinets. After 12 weeks of exposure, the paste cylinders were split and the carbonation front was visualized with the phenolphthalein colour indicator. In a next step, holes were drilled in the uncarbonated and carbonated zones of the fractured surfaces of cylinders. The powders collected were further crushed with mortar and pestle until all material could pass a sieve with a 74 µm mesh size. Per powder sample, around 50 mg was heated from 20°C to 1100°C at a rate of 10°C min^−1^ under an inert atmosphere (nitrogen). The mass change as a function of temperature was recorded with a Netzsch Sta 449 F3 Jupiter TGA apparatus. Small pieces of paste (Ø: 1–3 mm) that carbonated naturally in an atmosphere at 20°C and 60% RH, where the CO_2_ concentration is expected to be at around 0.03–0.04% CO_2_, were crushed and tested as well. Note that only one sample per test series was subjected to TGA. This is not a problem because we know from experience that the variation on the TGA outcome will be really small. Within the framework of a round robin test on hydration stoppage methods for phase assemblage of blended cements, duplicate TGA measurements were performed on uncarbonated pastes. The results of this round robin test have been published recently in Snellings *et al*. [14]. Analysis of the duplicate measurements for a cement-FA paste, for instance, indicated that the thermal decomposition curves almost coincided for the two replicates and that the scatter on the portlandite content was less than 1.2 g/100 g binder. Another recently published round robin study by Durdzinski *et al*. on degree of reaction of slag and FA in blended cements mentions a similar scatter of ±1.5 g/100 g binder [15].

The results of the thermogravimetric measurements were analysed in the Netzsch Proteus Analysis software [16]. To enable comparison between the OPC, HVFA and FA + SF pastes under investigation, the recorded mass losses during TGA were expressed in % relative to the residual mass of the powdery sample at 1100°C. Also note that the mass losses recorded during TGA were always corrected for the concurrent dehydration of other hydrated compounds. This was done in accordance with Baert [17].

For the determination of the Ca(OH)_2_ (CH) and CaCO_3_ (CC) content, a calculation method similar to the one suggested by Borges *et al*. [1] was used. The original method assumes that the presence of any carbonates in the unexposed paste samples as identified by means of TGA under an inert atmosphere solely originates from the initially available CH. In other words, the amount of additional carbonates that originate from any carbonate fractions present in the raw materials of the pastes was considered negligible. However, because the CO_2_ content of FA was observed to be almost twice the CO_2_ content of OPC, it was decided to correct for the mass loss (WL_CaCO_3_ original CO_2__) due to decarbonation of the original cement, FA and silica fume present in each paste (see tabulated CO_2_ percentages in table 2). Under this assumption, the initial CaCO_3_ content (%CC) could be calculated from the following equation:

2.3 $$\text{\%}CC_{\mathrm{unexposed}}=(WL_{\mathrm{CaC}O_{3}\;\mathrm{unexposed}}-WL_{\mathrm{CaC}O_{3\;}\mathrm{original}\;CO_{2}})\cdot \frac{MW_{\mathrm{CaC}O_{3}}}{MW_{CO_{2}}},$$

with WL_CaCO_3_ unexposed_, mass loss attributed to decarbonation of the unexposed paste, MW_CaCO_3__, molecular weight of CaCO_3_ (100 g mol^−1^) and MW_CO_2__, molecular weight of CO_2_ (44 g mol^−1^).

**Table 2.** Loss on ignition (%), Blaine fineness (m^2^ kg^−1^)/45 µm fineness (%)/BET surface area (m² g^−1^), density (kg m^−3^) and chemical composition (%) of the applied cement, fly ash and silica fume.

	CEM I 52.5 N	fly ash	silica fume
LOI	1.45	3.60	1.86
Blaine fineness	396.2	—	—
45 µm fineness	—	11.5	—
BET surface area	—	—	15.51
density	3137	2221	2232
CaO	62.77	3.56	0.20
SiO_2_	18.51	51.37	94.73
Al_2_O_3_	6.24	28.71	0.36
Fe_2_O_3_	4.12	5.10	0.71
MgO	1.08	1.01	0.39
K_2_O	0.64	1.77	0.90
Na_2_O	0.50	0.29	0.20
SO_3_	3.44	1.11	0.27
CO_2_	0.65	1.16	0.24
Cl^−^	—	0.001	—
free CaO	—	<0.1	—
reactive SiO_2_	—	37.48	—
Na_2_Oeq	—	1.46	—

The initial Ca(OH)_2_ content could be quantified by means of equation (2.4). This equation consists of two terms. The first term relates to the still present CH fraction in the paste sample and requires the mass loss due to decomposition of CH in CaO and H_2_O (WL_CH_) at around 400–500°C as input. The second term relates to the small CH fraction that had already carbonated during sample preparation and requires WL_CaCO_3_ unexposed_ as input. Further on, this equation depends on the molecular weights of CH (MW_CH_: 74 g mol^−1^), H_2_O (MW_H_2___O_: 18 g mol^−1^) and CO_2_ (MW_CO_2__: 44 g mol^−1^).

2.4 $$\text{\%}{\mathrm{CH}}_{\mathrm{unexposed}}={\mathrm{WL}}_{{\mathrm{CH}}_{\mathrm{unexposed}}}\cdot \frac{{\mathrm{MW}}_{\mathrm{CH}}}{{\mathrm{MW}}_{H_{2}O}}+({\mathrm{WL}}_{{\mathrm{CaCO}}_{3}\mathrm{unexposed}}-{\mathrm{WL}}_{{\mathrm{CaCO}}_{3}\;\mathrm{original}\;{\mathrm{CO}}_{2}})\cdot \frac{{\mathrm{MW}}_{\mathrm{CH}}}{{\mathrm{MW}}_{{\mathrm{CO}}_{2}}}.$$

The CC and CH contents of the intentionally carbonated pastes were not calculated in exactly the same way. For that purpose, equations (2.5) and (2.6) were used.

2.5 $$\text{\%}CC_{\mathrm{exposed}}=(WL_{\mathrm{CaC}O_{3}\;\mathrm{exposed}}-WL_{\mathrm{CaC}O_{3}\;\mathrm{original}\;CO_{2}})\cdot \frac{MW_{\mathrm{CaC}O_{3}}}{MW_{CO_{2}}}$$

2.6 $$\mathrm{and}\;\text{\%}CH_{\mathrm{exposed}}=WL_{\mathrm{CH}\;\mathrm{exposed}}\cdot \frac{MW_{\mathrm{CH}}}{MW_{H_{2}O}}.$$

Now, according to Borges *et al*. [1], the amount of C–S–H carbonation can be estimated indirectly from the CH and CC contents measured at the start and end of an accelerated carbonation experiment. To achieve this goal, the following sequence of calculations needs to be performed:

(i) Calculation of the %CH that carbonated before and during the carbonation experiment (A) by subtracting the amount of CH remaining after the test from the %CH that was initially available.
(ii) Calculation of the expected %CC that was formed out of the %CH that had carbonated (B) by multiplying the amount of carbonated CH (A) with MW_CaCO_3__/MW_Ca(OH)_2_._
(iii) Calculation of the estimated amount of carbonates that were the result of C–S–H carbonation (C) by subtracting the expected %CC that was formed out of the %CH that had carbonated (B) from the %CC that was measured at the end of the carbonation test.

This was the overall procedure followed by Borges *et al*. [1] to obtain an estimation of the amount of C–S–H carbonation for blended OPC–blast-furnace slag pastes when subjected to an accelerated carbonation test at the age of 90 days. The considered testing age is evidently very important when studying pastes containing pozzolanic SCMs such as FA and SF. After 90 days, the CH content of such a paste will not change much anymore because most of the pozzolanic hydration reactions have already taken place. However, this is not the case when an increased CO_2_ concentration was already applied after 28 days. At that moment, the CH content is still at its highest level because the pozzolanic reactions have not started yet or are still on-going. Thus, during exposure, the CH will be consumed not only by the (accelerated) carbonation reaction but also by the pozzolanic hydration reaction. As a consequence, when based on the initial CH content at the start of the carbonation experiment (cf. Borges *et al*. [1]), the estimated amount of C–S–H carbonation may be considerably underestimated. For this reason, it was decided to calculate (A), (B) and (C) also from the CH and CC contents of an unexposed paste sample with the same age (approx. 112 days) as the carbonated paste at the end of the test. The suggested alternative approach to estimate the amount of C–S–H carbonation will no longer underestimate the phenomenon. However, it still holds the risk of overestimating it because it is not known whether the carbonation reaction and the pozzolanic hydration reaction simply coexist without affecting each other. If the pozzolanic hydration reaction would be hindered due to the simultaneously occurring carbonation reaction, the SCMs may not hydrate to the same extent as they normally would in an unexposed paste. As such, the CH would be preferentially consumed by the carbonation reaction. This would result in more CH carbonation and less C–S–H carbonation. Being aware of the risks of under- or overestimating the amount of C–S–H carbonation depending on the procedure followed (the method of Borges *et al*. [1] or the suggested alternative method), the results of both methods were taken into consideration. The actual amount of C–S–H carbonation probably ranges between the boundaries set by the two calculation methods. For future experiments, it would be recommended though to also conduct a series of carbonation experiments starting at the age of 90 days when most of the pozzolanic hydration reactions have already taken place in order to make sure the observed amount of C–S–H carbonation does not relate to hydration of the pozzolans.

### 2.6. X-ray diffraction

The way of collecting and preparing the powdery samples for XRD analysis was very similar to the methodology described in the section on TGA (§2.5). The only difference in comparison with the latter was that a 10 m% ZnO internal standard was added to samples to enable absolute phase quantification in accordance with the Rietveld method for whole-powder pattern fitting. The powdery samples were side-loaded into the sample holder for analysis to reduce the effect of a preferential particle orientation. As such, the samples were inserted in a Thermo Scientific ARL X'tra Diffractometer equipped with a Peltier cooled detector and analysed in θ-2*θ* geometry over an angular range of 5–70° 2*θ* (Cu K radiation). The applied step size and counting time amounted to 0.02° 2*θ* and 1 s/step, respectively. All these settings were the same as in De Schepper *et al*. [18] and Snellings *et al*. [19]. Topas Academic V4.1 software was used for the Rietveld refinement [20]. The measurement specific or sample displacement error, a cosine Chebyshev function of 12 polynomial terms for background correction, the phase-specific scale factors, the unit cell parameters and the Lorentzian peak shape broadening parameters were among the refined parameters. Within the XRD patterns obtained, special attention was paid to the mineralogical phases that specifically relate to uncarbonated and carbonated samples, being portlandite, ettringite and kuzelite in the former condition and calcite, vaterite and aragonite in the latter condition. No correction was done for the variation in hydration degree between the powdery samples of the different paste mixtures during quantitative Rietveld analysis.

### 2.7. Mercury intrusion porosimetry

Possible changes in pore properties of the pastes that were exposed to 1% or 10% CO_2_ were studied using MIP. Little pieces of paste taken from the colorimetrically determined carbonated and uncarbonated zones of the paste cylinders were treated with liquid N_2_ and then stored in a LTE Scientific Mini Lyotrap freeze-dryer until their masses could be considered constant. The size of the samples did not exceed 14 mm in cross-section in order to make sure they could easily be inserted in the dilatometer of the MIP apparatus (PASCAL 140 + 440 Series, Thermo Fischer Scientific). For each analysis, around 1.3–1.4 g of sample was used. First, the samples were tested in the low pressure unit of the instrument (PASCAL 140 Series). There, the pressure on the mercury-filled dilatometer with the sample was gradually increased from 0 to 200 kPa. This intrusion phase was followed by an extrusion phase during which the pressure was lowered again to 100 kPa. Next, the dilatometer was moved to the high pressure unit of the MIP instrument (PASCAL 440 Series) where the samples were subjected to mercury pressures ranging from 0.1 to 200 MPa and back to 0.1 MPa. As such, the samples went again through an intrusion and an extrusion phase. Per type of sample, two replicates were tested. Afterwards, the results were analysed in the software application SOLiD [21]. The obtained pore size distributions and porosities corresponding with the capillary pores (0.01 µm < pore diameter D < 10 µm, cf. Gruyaert [22], St John *et al.* [23]) of completely carbonated paste after exposure to 1% and 10% CO_2_ as well as uncarbonated paste were compared with each other. Within the SOLiD software, the accessible porosity is calculated automatically by multiplying the total volume of intruded mercury during the test (in mm^3^ g^−1^, for pore diameters ranging from approximately 0.007 to 100 µm) with the samples' bulk density (which is also measured by the MIP apparatus during the test) in g mm^−3^. The capillary porosity was calculated in the same way, except for the fact that for this porosity only the capillary pore range (0.01 µm < pore diameter *D* < 10 µm) was considered.

## 3. Results

### 3.1. Effect on the carbonation coefficient/resistance

**Figure 1.**
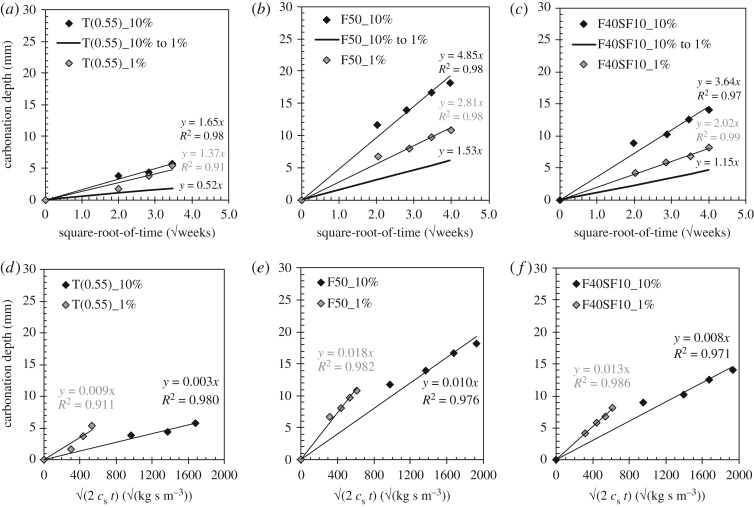
Effect of the applied CO_2_ concentration (10% versus 1% versus conversion from 10% to 1% cf. equation (2.1) on the carbonation coefficient (*a*,*b*,*c*) and the slope 1/√*R*_carb_ (*d*,*e*,*f*) of mixtures T(0.55), F50 and F40SF10.

Carbonation coefficients obtained after exposing the concrete to 10% CO_2_ are expected to be much higher than the carbonation coefficients of the same concrete after being exposed to only 1% CO_2_. In the case of the proposed HVFA and FA + SF concrete compositions, an obvious difference in carbonation rate could indeed be observed (figure 1). The OPC reference, however, showed a somewhat different behaviour. Exposure to only 1% CO_2_ still resulted in the lowest *A*_acce_ value (1.37 mm/√weeks). Nevertheless, the difference with the result obtained in a 10% CO_2_ atmosphere (1.65 mm/√weeks) is rather small.

One of the main reasons for performing these comparative carbonation tests was a validity check of the conversion formula to go from an accelerated to a field carbonation coefficient (equation (2.1)) for CO_2_ concentrations higher than 3% (§2.4). Therefore, the carbonation coefficients corresponding with exposure to 10% CO_2_ were implemented in this equation to estimate a carbonation coefficient for a 1% CO_2_ environment. Although seldom as high, perhaps only in industrial areas [10], the carbonation-related reaction products should not differ too much from the expected reaction products in a normal atmosphere at 0.03–0.04% CO_2_ [24].

If equation (2.1) would be valid, then the estimated carbonation coefficient for 1% CO_2_ should not differ too much from the measured value. This was clearly not the case for any of the considered concrete types. For mixtures F50, F40SF10 and T(0.55), the estimated values were only 55%, 57% and 38% of the measured values. In other words, the use of equation (2.1) results in an important underestimation of the field carbonation rate when based upon a carbonation test involving a much higher CO_2_ concentration (up to 10% CO_2_).

From the evaluation approach suggested by Visser [13], more or less the same conclusions can be drawn. Figure 1*d*–*f* clearly indicate that the √(1/*R*_carb_) slopes corresponding with a carbonation test at 10% CO_2_ significantly differ from the ones obtained at only 1%. Thus, increasing the CO_2_ concentration to 10% indeed induces non-negligible changes in comparison with the carbonation process at 1% CO_2_.

### 3.2. Effect on the chemistry of the carbonation reaction

In this section, the amount of CH and C–S–H carbonation was estimated after (accelerated) carbonation tests performed at different CO_2_ concentrations (0.03–0.04% CO_2_, 1% CO_2_ and 10% CO_2_). When calculated in accordance with Borges *et al*. [1], the CC and CH contents after 28 days at the start of the experiment together with CC and CH contents of the carbonated pastes at the end of the test (after 112 days) are to be used (table 3).

**Table 3.** Estimation of the extent of CH and C–S–H carbonation as calculated from TGA data (in g/100 g binder) obtained from pastes before and after carbonation, cf. Borges *et al*. [1].

before carbonation (at 28 days)	T(0.55)	F50	F40SF10						
CC initially present (equation (2.3))	0.0	0.0	1.3						
CH initially present (equation (2.4))	19.8	8.2	6.6						

After carbonation (at 112 days)	T(0.55)			F50			F40SF10		
CO_2_ concentration (%)	0.03–0.04	1	10	0.03–0.04	1	10	0.03–0.04	1	10
CC formed (equation (2.5))	54.5	71.0	68.5	23.2	30.5	35.0	28.9	32.4	35.2
CH remaining (equation (2.6))	0.0	0.0	0.0	0.5	0.0	0.0	0.0	0.0	0.0
Carbonated CH (A)	19.8	19.8	19.8	7.6	8.2	8.2	6.6	6.6	6.6
CC from carbonated CH (B)	26.8	26.8	26.8	10.3	11.0	11.0	8.9	8.9	8.9
CC from C–S–H carbonation (C)	27.7	44.2	41.7	12.9	19.5	23.9	19.9	23.5	26.2
%CC from carbonated CH	49	38	39	44	36	32	31	27	26
%CC from C–S–H carbonation	51	62	61	56	64	68	69	73	74

(A) = %CH initially available (equation (2.4)) – %CH remaining (equation (2.6)).

(B) = (A)×MW_CaCO_3__/MW_Ca(OH)_2_._

(C) = %CC formed (cf. equation (2.5)) – (B).

As such, the highest percentages of C–S–H carbonation were recorded for the FA + SF paste (69–74%). In the HVFA paste, these percentages were slightly lower (56–68%), while the lowest percentages of C–S–H carbonation were observed for the OPC paste (51–62%). However, the differences between the OPC and HVFA pastes were actually not that pronounced. The applied CO_2_ concentration during carbonation testing also seems to play a role. For every studied paste mixture, the estimated amount of C–S–H carbonation increased with increasing CO_2_ concentration. The most substantial difference was seen between the pastes that carbonated naturally at 0.03–0.04% CO_2_ and the pastes that were subjected to an accelerated carbonation test either at 1% or 10% CO_2_. The differences in amount of C–S–H carbonation between the latter two test conditions were rather negligible (1–4%).

As already mentioned in §2.5, this approach probably underestimates the actual amount of C–S–H carbonation for the pastes with FA (and SF) because the pozzolanic reactions have not started yet or are still on-going after only 28 days. The alternative calculation method does not depend on the amount of CC and CH present at the start of the carbonation test, but on the corresponding amounts present in unexposed pastes with the same age as the carbonated pastes at the end of the test (after 112 days). Except for the fact that the as such obtained percentages of C–S–H carbonation for paste mixtures F50 and F40SF10 are higher (table 4), basically all previously drawn conclusions remain valid.

**Table 4.** Estimation of the extent of CH and C–S–H carbonation as calculated from TGA data (in g/100 g binder) obtained from uncarbonated and carbonated pastes at the age of 112 days.

uncarbonated (at 112 days)	T(0.55)	F50	F40SF10						
CC normally present (equation (2.3))	0.0	0.1	0.0						
CH normally present (equation (2.4))	19.0	4.3	4.6						

carbonated (at 112 days)	T(0.55)			F50			F40SF10		
applied CO_2_ concentration (%)	0.03–0.04	1	10	0.03–0.04	1	10	0.03–0.04	1	10
CC formed (equation (2.5))	54.5	71.0	68.5	23.2	30.5	35.0	28.9	32.4	35.2
CH remaining (equation (2.6))	0.0	0.0	0.0	0.5	0.0	0.0	0.0	0.0	0.0
carbonated CH (A)	19.0	19.0	19.0	3.8	4.3	4.3	4.6	4.6	4.6
CC from carbonated CH (B)	25.7	25.7	25.7	5.1	5.8	5.8	6.3	6.3	6.3
CC from C–S–H carbonation (C)	28.8	45.3	42.8	18.1	24.7	29.1	22.6	26.1	28.9
%CC from carbonated CH	47	36	37	22	19	17	22	19	18
%CC from C–S–H carbonation	53	64	63	78	81	83	78	81	82

(A) = %CH normally present (equation (2.4)) – %CH remaining (equation (2.6)).

(B) = (A)×MW_CaCO_3__/MW_Ca(OH)_2__.

(C) = %CC formed (cf. equation (2.5)) – (B).

If the paste contained FA (and SF), more C–S–H carbonation is observed than when the binder fraction only consisted of OPC. Regarding the applied CO_2_ concentration, the most notable difference in amount of C–S–H carbonation (3–5%) again existed between the natural and accelerated carbonation conditions. For the OPC paste, a difference of up to 10% was recorded. The differences in amount of C–S–H carbonation after exposure to 1% or 10% CO_2_ were insignificant (1%) for all pastes considered. The C–S–H carbonation percentages obtained with the alternative calculation method may be somewhat exaggerated for the pastes containing pozzolans. While the method of Borges *et al*. [1] underestimates C–S–H carbonation, the alternative method may overestimate it. The actual amounts of carbonates that originate from the C–S–H will range between the outcomes of the two methods. However, the trends observed with each method will probably remain.

Finally, it should be noted that only a TGA-based calculation of the amount of C–S–H carbonation revealed an influence of the applied CO_2_ concentration during carbonation testing. No influence could be detected from a mere analysis of the most basic TGA output, i.e. the thermal decompositions curves of each sample. In contrast with the findings of Thiéry *et al*. [5] the decarbonation-related mass losses did not occur in different temperature ranges depending on the type of calcium carbonate that is decomposing (550–680°C: the amorphous phase; 680–780°C: the metastable phase, being vaterite and aragonite; 780–990°C: the stable phase, being well-crystallized calcite). Usually, the thermal decomposition curves of carbonated samples showed a continuous mass loss between 550°C and 990°C.

### 3.3. Effect on the mineralogical phases formed

Quantitative Rietveld analysis was done on the X-ray diffractograms of the OPC, HVFA and FA + SF pastes which are included in the electronic supplementary material to this paper. The results are shown in tables 5–7. Attention was especially paid to the mineralogical phases that are normally present when uncarbonated (portlandite, kuzelite, ettringite) and carbonated (calcite, vaterite, aragonite). Apart from those, some of the other phases measured specifically relate to one or more of the binder materials used, e.g. OPC (β-C_2_S, C_4_AF, hydrogarnet), FA and SF (mullite, quartz_low). The percentages mentioned for **‘**other’ comprise crystalline material present in amounts smaller than the detection limit of the apparatus as well as non-crystalline material. Tables 5–7 give three analyses for the pastes that were fully carbonated at 10% CO_2_. Samples 10%a, 10%b and 10%c were collected from the holes drilled 5–7 mm, 10–12 mm and 18–22 mm from the exposed mantle surfaces of the paste cylinders, respectively.

**Table 5.** Quantitative Rietveld analysis of OPC paste T(0.55) in uncarbonated condition (N) and after carbonation at 0.03–0.04%, 1% and 10% CO_2_.

phase (%)	N	0.03–0.04%	1%	10%a	10%b	10%c
β-C_2_S	6.9 ± 0.3	2.9 ± 0.3	2.1 ± 0.3	2.0 ± 0.4	2.4 ± 0.4	1.9 ± 0.4
C_4_AF	3.6 ± 0.2	3.7 ± 0.2	4.0 ± 0.3	4.3 ± 0.3	3.9 ± 0.3	3.4 ± 0.3
quartz_low	—	—	—	—	—	—
mullite	—	—	—	—	—	—
portlandite	18.5 ± 0.4	2.7 ± 0.3	0.4 ± 0.4	0.3 ± 0.4	0.1 ± 0.6	0.1 ± 0.5
kuzelite	1.4 ± 0.2	—	—	—	—	—
ettringite	3.0 ± 0.2	0.7 ± 0.2	0.4 ± 0.3	0.4 ± 0.3	0.6 ± 0.3	—
hydrogarnet	0.7 ± 0.2	0.5 ± 0.2	0.8 ± 0.2	0.7 ± 0.3	0.8 ± 0.2	0.7 ± 0.2
calcite	1.8 ± 0.2	4.1 ± 0.2	4.0 ± 0.2	5.6 ± 0.3	6.2 ± 0.3	6.2 ± 0.3
vaterite	2.3 ± 0.3	29.6 ± 0.5	36.9 ± 0.6	41.2 ± 0.6	40.4 ± 0.7	36.2 ± 0.6
aragonite	—	2.6 ± 0.3	—	—	—	—
other	61.9 ± 0.7	53.2 ± 0.8	51.4 ± 0.9	45.5 ± 1.0	45.6 ± 1.1	51.4 ± 1.0
calcite + vaterite + aragonite	4.1 ± 0.3	36.3 ± 0.6	41.0 ± 0.6	46.8 ± 0.7	46.6 ± 0.7	42.4 ± 0.7
calcite/(vaterite + aragonite)	0.80 ± 0.12	0.13 ± 0.01	0.11 ± 0.01	0.13 ± 0.01	0.15 ± 0.01	0.17 ± 0.01

**Table 6.** Quantitative Rietveld analysis of HVFA paste F50 in uncarbonated condition (N) and after carbonation at 0.03–0.04%, 1% and 10% CO_2_.

phase (%)	N	0.03–0.04%	1%	10%a	10%b	10%c
β-C_2_S	2.7 ± 0.3	1.9 ± 0.2	1.6 ± 0.3	1.5 ± 0.3	1.4 ± 0.2	1.5 ± 0.2
C_4_AF	1.1 ± 0.2	1.6 ± 0.2	1.9 ± 0.2	1.6 ± 0.2	1.6 ± 0.2	1.5 ± 0.2
quartz_low	4.0 ± 0.1	3.2 ± 0.1	4.9 ± 0.2	3.7 ± 0.2	3.7 ± 0.2	3.6 ± 0.2
mullite	7.4 ± 0.3	8.0 ± 0.3	9.3 ± 0.3	7.8 ± 0.3	8.3 ± 0.3	8.4 ± 0.3
portlandite	4.6 ± 0.2	0.6 ± 0.3	0.1 ± 0.3	—	—	0.1 ± 0.2
kuzelite	1.4 ± 0.1	0.3 ± 0.1	0.1 ± 0.1	0.1 ± 0.1	0.2 ± 0.1	—
ettringite	1.1 ± 0.2	0.2 ± 0.2	0.5 ± 0.2	0.2 ± 0.2	0.4 ± 0.2	0.2 ± 0.2
hydrogarnet	0.4 ± 0.2	0.4 ± 0.2	0.5 ± 0.2	0.4 ± 0.2	0.4 ± 0.2	0.2 ± 0.2
calcite	0.5 ± 0.1	2.7 ± 0.2	1.0 ± 0.1	3.2 ± 0.2	2.0 ± 0.1	2.2 ± 0.1
vaterite	—	17.1 ± 0.4	20.9 ± 0.4	19.4 ± 0.4	20.2 ± 0.4	19.7 ± 0.4
aragonite	—	0.4 ± 0.2	0.4 ± 0.2	0.2 ± 0.2	0.1 ± 0.2	0.1 ± 0.2
other	76.7 ± 0.5	63.7 ± 0.8	58.7 ± 0.8	62.0 ± 0.8	61.6 ± 0.8	62.5 ± 0.7
calcite + vaterite + aragonite	0.5 ± 0.1	20.2 ± 0.5	22.4 ± 0.5	22.7 ± 0.5	22.2 ± 0.5	22.0 ± 0.5
calcite/(vaterite + aragonite)	—	0.16 ± 0.01	0.05 ± 0.01	0.16 ± 0.01	0.10 ± 0.01	0.11 ± 0.01

**Table 7.** Quantitative Rietveld analysis of FA + SF paste F40SF10 in the uncarbonated condition (N) and after carbonation at 0.03–0.04%, 1% and 10% CO_2_.

phase (%)	N	0.03–0.04%	1%	10%a	10%b	10%c
β-C_2_S	3.2 ± 0.3	1.6 ± 0.2	2.0 ± 0.2	0.4 ± 0.2	0.5 ± 0.2	0.8 ± 0.2
C_4_AF	1.1 ± 0.2	1.7 ± 0.2	1.7 ± 0.2	1.2 ± 0.2	1.4 ± 0.2	1.3 ± 0.1
quartz_low	2.8 ± 0.1	2.8 ± 0.1	3.2 ± 0.1	2.6 ± 0.1	2.8 ± 0.1	2.6 ± 0.1
mullite	6.5 ± 0.3	6.8 ± 0.3	6.9 ± 0.3	6.9 ± 0.3	6.8 ± 0.3	6.1 ± 0.3
portlandite	5.7 ± 0.2	0.3 ± 0.2	0.1 ± 0.3	0.1 ± 0.2	—	—
kuzelite	0.2 ± 0.1	0.1 ± 0.1	—	0.1 ± 0.1	0.1 ± 0.1	0.1 ± 0.1
ettringite	2.3 ± 0.2	0.5 ± 0.2	0.3 ± 0.2	0.2 ± 0.2	0.4 ± 0.2	0.4 ± 0.2
hydrogarnet	0.3 ± 0.2	0.3 ± 0.2	0.3 ± 0.2	0.3 ± 0.2	–	0.1 ± 0.2
calcite	0.8 ± 0.1	1.8 ± 0.1	1.8 ± 0.1	11.5 ± 0.3	7.3 ± 0.2	6.3 ± 0.2
vaterite	—	16.4 ± 0.3	15.6 ± 0.3	7.7 ± 0.3	13.7 ± 0.3	14.3 ± 0.3
aragonite	—	0.1 ± 0.2	1.8 ± 0.2	0.1 ± 0.2	0.3 ± 0.2	0.3 ± 0.2
other	77.2 ± 0.6	67.6 ± 0.7	66.3 ± 0.7	69.0 ± 0.7	66.6 ± 0.8	67.8 ± 0.7
calcite + vaterite + aragonite	0.8 ± 0.1	18.3 ± 0.4	19.2 ± 0.4	19.3 ± 0.5	21.3 ± 0.5	20.9 ± 0.4
calcite/(vaterite + aragonite)	—	0.11 ± 0.01	0.10 ± 0.01	1.47 ± 0.08	0.52 ± 0.02	0.44 ± 0.02

From the quantitative assessment of the mineralogical phases present in OPC paste T(0.55), the following conclusions can be drawn:

— As the entire binder content consisted of OPC, β-C_2_S, C_4_AF and hydrogarnet were the only binder-related phases detected. Quartz_low and mullite were not present. In uncarbonated condition, the β-C_2_S content exceeded the C_4_AF content. After carbonation, quite the opposite was true.
— In uncarbonated condition, the paste logically contains a substantial amount (18.5 ± 0.4%) of portlandite (Ca(OH)_2_), a main hydration product of the cement. Kuzelite and ettringite were also present, be it in much smaller amounts (1.4 ± 0.2% and 3.0 ± 0.2%, respectively).
— Small amounts of calcite (1.8 ± 0.2%) and vaterite (2.3 ± 0.3%) were measured in the unexposed condition. Their presence is attributed to a slight carbonation of the samples during their preparation in a non-CO_2_-free environment.
— When carbonated, the remaining amount of portlandite decreased with an increasing CO_2_ concentration. This amount became negligible starting from a CO_2_ concentration of 1%. The same goes for the ettringite content. The kuzelite fraction already completely disappears starting from a CO_2_ concentration of 0.03–0.04%.
— After carbonation, the metastable vaterite phase was most abundantly available (29.6–41.2%). This statement holds true for every CO_2_ concentration considered. Carbonation at 1% and 10% CO_2_ resulted in the highest values. The fraction of stable calcite was much smaller (4.0–6.2%). Somewhat higher percentages were recorded after carbonation at 10% CO_2_. The metastable aragonite was measured only in the naturally carbonated paste at 0.03–0.04% CO_2_. The calcite + vaterite + aragonite contents ranged 36.3–46.8%. The highest percentages were recorded after exposure to 10% CO_2_.
— When considering the calcite/(vaterite + aragonite) ratio, no important changes with increasing CO_2_ concentration could be observed. Their values ranged 0.11–0.17 which was much lower when compared with the uncarbonated sample (0.80).

An in-depth analysis of the results obtained for the HVFA paste F50 led to the following conclusions:

— As 50% of the binder system in this paste consisted of FA, a shift in the presence of the binder-related phases was clearly visible. Substantial amounts of quartz_low (3.2–4.9%) and mullite (7.4–9.3%) were now detected, while the β-C_2_S and the C_4_AF were lower. In uncarbonated condition, the β-C_2_S content again exceeded the C_4_AF content. After carbonation, both contents were more or less similar, which means that mainly the β-C_2_S content was affected by carbonation. The low hydrogarnet fraction remained stable.
— In uncarbonated condition, the portlandite content (4.6 ± 0.2%) of the HVFA paste is obviously lower than the portlandite content of the OPC paste (18.5 ± 0.4%) because the paste contains 50% less OPC that can hydrate to form portlandite. Moreover, the reduced amount of portlandite formed is consumed by the pozzolanic FA reaction. The measured percentages of kuzelite and ettringite were also much lower (1.4 ± 0.1% and 1.1 ± 0.2%, respectively).
— After natural (0.03–0.04% CO_2_) and accelerated (1% and 10% CO_2_) carbonation, the remaining amounts of portlandite, kuzelite and ettringite became negligible.
— The carbonation reaction products mainly consisted of metastable vaterite (17.1–20.9%). Much less stable calcite was present (1.0–3.2%). The amounts of aragonite were negligible. No obvious effect of the applied CO_2_ concentration could be observed, also when the calcite + vaterite + aragonite contents (20.2–22.7%) and the ratio of the stable and metastable phases (0.05–0.16) were taken into consideration. The total amounts of carbonation reaction products were found to be lower than the corresponding amounts that were recorded for the OPC paste (36.3–46.8%). This was no surprise because there was considerably less portlandite available for the carbonation reaction.

The main findings of the Rietveld analyses performed on the FA + SF pastes were more or less similar:

— Again smaller amounts of β-C_2_S (3.2 ± 0.3%) and C_4_AF (1.1 ± 0.2%) were present when uncarbonated. After carbonation, mainly the β-C_2_S content decreased. After carbonation at 10% CO_2_, the β-C_2_S phase almost completely disappeared. This phenomenon was not observed for the HVFA paste. In comparison with the same HVFA paste, quartz_low and mullite were present in somewhat smaller amounts. Their contents remained stable at 2.6–3.2% and 6.1–6.9%, respectively, regardless of the carbonation condition of the sample. A low hydrogarnet fraction was present which was not affected by carbonation.
— In uncarbonated condition, the portlandite content (5.7 ± 0.2%) was again much lower in comparison with OPC paste, because of the presence of 50% less OPC that can hydrate to form portlandite and because of the portlandite consuming pozzolanic reactions of both the FA and the silica fume. Small amounts of kuzelite and ettringite were noted as well. The latter two phases almost completely disappeared after carbonation at any of the studied CO_2_ concentrations.
— The total content of carbonation reaction products was similar for all the CO_2_ concentrations that were studied and was found to be slightly lower than the corresponding values of the HVFA paste. The major carbonation reaction product was again vaterite (13.7–16.4%). There was one exception though. The sample carbonated at 10% which was collected 5–7 mm from the exposed surface (10%a) was characterized by the vaterite content of only 7.7 ± 0.3%, while the calcite content was no less than 11.5 ± 0.3%. For the moment, this deviating behaviour cannot be explained. Further research on this matter is imperative. The calcite content seems to depend very much on the applied CO_2_ concentration. For low CO_2_ concentrations (0.03–0.04% and 1%), the calcite content after carbonation is very small (1.8 ± 0.1%). Substantially higher calcite percentages were recorded after carbonation at 10%. This difference is especially visible when the calcite/(vaterite + aragonite) ratio is considered (0.03–0.04% to 1%: 0.10–0.11 versus 10%: 0.44–1.47). As such, mixture F40SF10 was the paste composition that was most obviously affected by the applied CO_2_ concentration during carbonation testing. The OPC and HVFA pastes were barely influenced by this parameter. However, it should be noted that this conclusion is based on the XRD measurements only. There seems to be no link with the estimated amounts of C–S–H carbonation as calculated from the TGA output.

### 3.4. Effect on the porosity and pore size distribution

**Figure 2.**
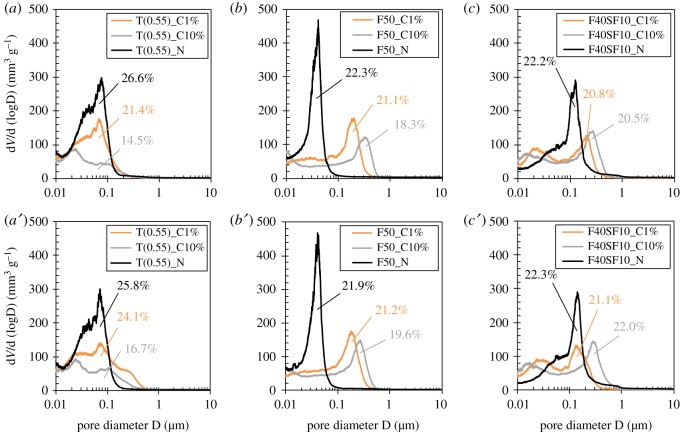
Pore size distributions for the capillary pores and capillary porosities (%) of two replicates of each paste studied (T(0.55): (a, a’); F50: (b, b'); F40SF10: (c, c') in uncarbonated condition (N) and after carbonation at 1% and 10% CO_2_.

Pore size distributions for the capillary pore range (0.01 µm < *D* < 10 µm) and the corresponding capillary porosities of OPC paste T(0.55), HVFA paste F50 and FA + SF paste F40SF10 are presented in figure 2.

The following trends were observed:

— Carbonation at 1% and 10% CO_2_ induces a pronounced reduction in capillary porosity of the OPC paste. This reduction was most striking after exposure to the highest CO_2_ concentration. When uncarbonated, this porosity amounted to 25.8–26.6%. Carbonation at 1% CO_2_ made this porosity decrease to 21.4–24.1%. After carbonation at 10% CO_2_, this porosity equalled only 14.5–16.7%. There was also a shift for the major peak in the pore size distribution. When uncarbonated or carbonated at 1% CO_2_, this peak corresponded with a pore diameter of around 0.07 µm. Exposure to 10% CO_2_ induced a shift of this peak to a pore diameter of only a little more than 0.02 µm, which indicates that the high CO_2_ concentration imposed caused serious densification of the pore structure which is normally induced by CH carbonation. The presence of more metastable vaterite with a higher solubility than calcite, a phase which was present in much smaller amounts, did not seem to affect this CH carbonation-induced densification in a negative way (§3.3). Moreover, it should be noted that the amount of CH carbonation in the pastes carbonated at 1% and 10% CO_2_ was rather similar (§3.2). Thus, the increased porosity reduction and pore structure densification of OPC paste with an increasing CO_2_ concentration cannot simply be explained by the fact that more CH was carbonated. However, the MIP results for OPC paste could explain why a carbonation coefficient for 1% CO_2_ estimated from a carbonation experiment conducted at 10% CO_2_ using equation (2.1) is so much lower than a carbonation coefficient measured during a carbonation experiment performed at 1% CO_2_.
— Carbonation induces a limited reduction in capillary porosity for HVFA paste. While an uncarbonated sample could be characterized by a capillary porosity of 21.9–22.3%, samples carbonated at 1% and 10% CO_2_ had a capillary porosity of 21.1–21.2% and 18.3–19.6%, respectively. There is also an obvious difference between two CO_2_ concentrations considered for accelerated carbonation testing. Carbonation caused a slight coarsening of the pore structure. This could be concluded from the peak shift in the pore size distribution from a pore diameter of 0.04 µm in uncarbonated condition towards a pore diameter of 0.2 µm and 0.3 µm after carbonation at 1% and 10% CO_2_. A higher CO_2_ concentration seems to have a stimulating effect on the coarsening of the pore structure. As the latter phenomenon is usually attributed to C–S–H carbonation, one would expect a bit more C–S–H carbonation after carbonation at 10% CO_2_ instead of at 1% CO_2_. This was indeed the case. However, the observed differences seemed rather negligible (§3.2) and thus cannot fully explain an increased coarsening with an increasing CO_2_ concentration. In the perspective of the estimated carbonation coefficients from both experiments, the lower values that result from the highly accelerated test using equation (2.1) (§3.1) can only be supported by its lower capillary porosity and not by the fact that this porosity consisted of larger pores.
— The capillary porosities of the FA + SF paste (22.2–22.3%) in uncarbonated condition were similar to those recorded for the HVFA paste. The main pore diameter (0.15 µm) was considerably larger though (F50: 0.04 µm). This coarser pore structure is rather surprising given the presence of 10% silica fume and the higher strength class of the corresponding concrete (table 1: C50/60 versus C40/50). The underlying causes certainly need to be investigated further on, especially in relation to the expected similar carbonation behaviour of paste and concrete as reported by Hermida *et al*. [9] and their related pore structure.
— Carbonation of the F40SF10 paste induced only a slight reduction in capillary porosity. The differences in porosity after carbonation at 1% and 10% CO_2_ (20.8–21.1% versus 20.5–22.0%) were not very pronounced. Carbonation induced a coarsening of the pore structure again. Carbonation at 1% and 10% CO_2_ induced a shift of the main pore diameter to 0.2 µm and 0.3 µm. Similar shifts were observed for the HVFA paste. The fact that just slightly more C–S–H carbonated at 10% CO_2_ (§3.2) seems not enough to explain the shift to 0.3 µm instead of 0.2 µm. An important difference with the HVFA paste is the occurrence of second smaller peak in the pore size distribution for the capillary pore range. These peaks correspond with very small pore diameters, i.e. 0.025 µm at 1% CO_2_ and 0.015 µm at 10% CO_2_. Their occurrence implicates that there was, to some extent, a densification of the pore structure. Now, a similar capillary porosity consisting mainly of larger pores (0.3 µm) and only to some extent of very fine pores (0.015 µm) cannot explain the lower carbonation rates estimated from a carbonation experiment at 10% CO_2_ as opposed to a carbonation coefficient measured during exposure to just 1% CO_2_.

## 4. Discussion

So far, neither of the applied investigation techniques (TGA, XRD and MIP) applied on uncarbonated and carbonated samples in this study could reveal the actual cause of the underestimation of the estimated carbonation coefficients for 1% CO_2_ when based on a highly accelerated carbonation test involving exposure to 10% CO_2_. True, the observed porosity reduction and strong densification of the pore structure of OPC paste after carbonation at 10% could explain this to some extent, yet not completely. For the HVFA and FA + SF pastes, the explanation must be sought elsewhere because these binder systems behaved in a totally different way. Probably, the available conversion formula to go from an accelerated to a field carbonation coefficient (equation (2.1)) is simply not entirely correct. Further research on its improvement is therefore imperative. One of the theories that most certainly must be verified further on is the excessive production of water during carbonation at 10% CO_2_ which induces pore blocking, cf. Saetta and Vitaliani [25]. The carbonation-induced production of calcium carbonate always coincides with the release of water. When carbonating concrete at a high CO_2_ concentration, the amount of water produced could be more than the porous matrix is capable of expelling in the same time interval. The time needed to establish a condition of equilibrium again is believed to slow down the propagation of the carbonation depth [10,25]. Da Silva *et al*. also mention that CO_2_ solubility is low when high CO_2_ concentrations are used [10]. The penetrating CO_2_ first needs to transform into acid in the presence of water before the actual carbonation reaction can take place and the amount of CO_2_ capable of dissolving in water is limited. If these mechanisms would turn out more dominant than the coarsening of the pore structure due to the carbonation shrinkage attributable to the acceleration of the carbonation test, applying a high CO_2_ concentration would underestimate the carbonation depth and rate under field conditions. Further experiments are on-going to evaluate the contribution of the mechanisms related to carbonation-induced water release. Recent results of neutron radiography experiments and monitoring of HVFA mortars with embedded humidity sensors and multiring electrodes during accelerated carbonation indeed seem to imply that these mechanisms cannot be neglected [26,27].

## 5. Conclusion

When carbonation rates for 1% CO_2_ are estimated from an accelerated carbonation experiment at 10% CO_2_ using the conversion formula of Sisomphon & Franke [12], these rates are by far lower than the ones measured at 1% CO_2_ for HVFA, FA + SF and OPC concrete. The underestimation inherent to carbonation testing at 10% CO_2_ cannot simply be explained by an important reduction in porosity and densification of the pore structure as assessed by means of MIP. But, TGA and XRD analyses indicate that the carbonation mechanisms during exposure to 1% and 10% CO_2_ are similar. More important differences exist between natural carbonation at 0.03–0.04% CO_2_ and slightly accelerated carbonation at 1% CO_2_. In that perspective, it would be worthwhile to also perform a series of natural carbonation tests at 0.03–0.04% CO_2_ and see whether conversion of the carbonation rates obtained at 1% CO_2_ to values representative for 0.03–0.04% CO_2_ really match with the ones actually measured at 0.03–0.04% CO_2_. Recently published results seem to indicate that there is still a slight underestimation when field carbonation rates are estimated from accelerated carbonation tests at 1% CO_2_ [27]. Still more research is needed to come up with a more accurate conversion formula. Probably, such a formula will also need to account for other relevant mechanisms involved, for instance possible pore blocking induced by excess water production during carbonation at higher CO_2_ levels.

## Supplementary Material

XRD diffractograms

## Data accessibility

The datasets supporting this article have been uploaded as part of the electronic supplementary material.
