# Supplementary material for: Accelerated and natural carbonation of concrete with high volumes of fly ash: chemical, mineralogical and microstructural effects
Source: R Soc Open Sci. 2019 Jan 16;6(1):181665. doi: 10.1098/rsos.181665 (PMC6366180; doi:10.1098/rsos.181665)
Supplement: XRD diffractograms [file rsos181665supp1.docx]

**Supplementary material – X-ray diffractograms**

Figure S.1. X-ray diffractogram of OPC paste T(0.55) in uncarbonated condition (N) and after carbonation at 0.03-0.04%, 1% and 10% CO_2_.

Figure S.2. X-ray diffractogram of HVFA paste F50 in uncarbonated condition (N) and after carbonation at 0.03-0.04%, 1% and 10% CO_2_.

Figure S.3. X-ray diffractogram of FA+SF paste F40SF10 in uncarbonated condition (N) and after carbonation at 0.03-0.04%, 1% and 10% CO_2_.
